# Cost-Effectiveness of Sequential Denosumab/Zoledronic Acid Compared With Zoledronic Acid Monotherapy for Postmenopausal Osteoporotic Women in China

**DOI:** 10.3389/fphar.2022.816248

**Published:** 2022-03-18

**Authors:** Ruxu You, Jinyu Liu, Lei Ke, Min Wan, Yu Zhang, Guangyi Yu, Takahiro Mori

**Affiliations:** ^1^ Department of Pharmacy, Union Hospital, Tongji Medical College, Huazhong University of Science and Technology, Wuhan, China; ^2^ Department of Pharmacy, Tongji Hospital, Tongji Medical College, Huazhong University of Science and Technology, Wuhan, China; ^3^ Department of Pharmacy, People’s Hospital of Dongxihu District, Wuhan, China; ^4^ Department of General Medical Science, Graduate School of Medicine, Chiba University, Chiba, Japan; ^5^ Health Services Research and Development Center, University of Tsukuba, Tsukuba, Japan; ^6^ Department of General Internal Medicine, Eastern Chiba Medical Center, Togane, Japan

**Keywords:** postmenopausal osteoporosis, sequential therapy, denosumab, zoledronic acid, cost-effectiveness analysis

## Abstract

**Objective:** The primary purpose of this study was to estimate the cost-effectiveness of sequential denosumab/zoledronic acid versus zoledronic acid monotherapy for postmenopausal osteoporotic women in China.

**Methods:** We updated and utilized a previously validated Markov microsimulation model to obtain the cost-effectiveness of two strategies for treating postmenopausal osteoporotic women. We compared the incremental cost-effectiveness ratios (ICERs) (US dollars [$] per quality-adjusted life year [QALY]) of sequential denosumab/zoledronic acid (i.e., biannual subcutaneous denosumab for 3 years followed by annual intravenous zoledronic acid for 3 years) with zoledronic acid monotherapy for 3 years in Chinese women with postmenopausal osteoporosis at ages 65, 70, 75, and 80 from the health care payer perspective. Our study’s willingness-to-pay (WTP) threshold was set to three times the value of China’s per capita GDP in 2020 ($31,512).

**Results:** The ICERs of sequential denosumab/zoledronic acid versus zoledronic acid monotherapy were $59,389/QALY, $23,821/QALY, $22,710/QALY, and $14,027/QALY at age 65, 70, 75, and 80 years, respectively. One-way sensitivity analyses showed that the most impactful parameter was the persistence rate of the medications. If the persistence rate of denosumab or zoledronic acid was increased by 10%, sequential denosumab/zoledronic acid would be cost-effective at age 65. In probabilistic sensitivity analyses, the probabilities of sequential denosumab/zoledronic being cost-effective compared to zoledronic acid monotherapy were approximately 29.3%, 68.7%, 86.1%, and 99.4% for ages 65, 70, 75, and 80 years, respectively, at the WTP threshold of $31,512/QALY.

**Conclusion:** Among Chinese postmenopausal osteoporosis women over 70 years old, sequential denosumab/zoledronic acid was cost-effective compared with zoledronic acid monotherapy at the pre-determined WTP threshold.

## Introduction

Osteoporosis is a metabolic skeletal disease characterized by decreased bone mass and destruction of bone tissue microstructure. Osteoporosis is associated with a raised risk of bone fragility and leads to significant morbidity and mortality (Cosman et al., 2017; Katsoulis et al., 2017; Melton et al., 2014; Wang et al., 2009). Epidemiological surveys showed that the prevalence of osteoporosis among people over the age of 65 reached 32.0% in China (Si et al., 2015; Liu et al., 2018; Chinese Society of Osteoporosis and Bone Mineral Research, 2019). More than 50% of osteoporotic fractures will appear in Asia by 2050, according to the calculation of the International Osteoporosis Foundation (IOF), and China will be the most seriously affected country due to the huge number of the elderly population (over 176 million aged >65 years in 2020) (Pisani et al., 2016). Osteoporosis can lead to fragility fractures (e.g., hip, spine, and wrist), which significantly affect patients’ quality of life and limits their daily activities. Furthermore, in previous studies, the costs related to osteoporosis in China were projected to reach US$25.6 billion by 2050, which will mean a heavy health care socio-economic burden (Si et al., 2015; Liu et al., 2018).

There are multiple pharmacological treatment options for osteoporosis. Bisphosphonates such as oral alendronate are classic anti-osteoporosis drugs that inhibit bone resorption. Due to poor persistence and compliance with oral medications, intravenous zoledronic acid once a year has become a popular choice among bisphosphonates for treating osteoporosis in postmenopausal women. Although bisphosphonates have shown significant anti-osteoporosis benefits, there are still risks that limit a long-term use. The potential dangers of long-term use of bisphosphonates include osteonecrosis of the jaw and atypical femoral fractures (Sellmeyer, 2010; Adler et al., 2016).

With the continuous advancement of biomedicine, drugs with different action mechanisms have emerged in succession. Denosumab is a fully humanized monoclonal antibody specific to the receptor activator of nuclear factor-kappa B ligand (RANKL), which can inhibit the binding of RANKL to its receptor RANK and reduce the formation, function, and survival of osteoclasts. Based on research at home and abroad, denosumab has been proven to significantly reduce the risk of hip, vertebral, and non-vertebral fractures in postmenopausal women (Parthan et al., 2014; Y.; Wang et al., 2009). However, recent studies have indicated that, in contrast to those receiving bisphosphonates, patients receiving denosumab should not withdraw their medication after a given treatment period because discontinuing denosumab may increase the risk of vertebral fractures (Anastasilakis & Makras, 2016; Popp et al., 2016; Fernandez Fernandez et al., 2020). Therefore, current studies have increasingly mentioned the importance of sequential treatments following denosumab with anti-osteoporosis drugs of different mechanisms (Mori et al., 2017a; Mori et al., 2017b; Eastell et al., 2019). Bisphosphonates are usually prescribed after other anti-osteoporosis treatments to prevent bone density decline and fracture efficacy loss (Feng et al., 2013). However, the choice of treatment should take safety, effectiveness, economy, and other patient-related factors into consideration.

To the best of our knowledge, the economic evaluations of sequential treatment of denosumab and zoledronic acid have not been reported on postmenopausal osteoporosis women in China. The primary purpose of this analysis was to analyze the pharmacoeconomics information of sequential denosumab/zoledronic acid versus zoledronic acid monotherapy from the perspective of Chinese health care payer.

## Methods

### Overview

A previously validated Markov microsimulation model (You et al., 2020; You & Liu, 2020) was updated and made available for assessing the cost-effectiveness of sequential denosumab/zoledronic acid compared with zoledronic acid monotherapy and with no treatment. The target population was Chinese postmenopausal women with no history of hip, vertebral, or wrist fracture at four various ages (65, 70, 75, and 80) of treatment initiation. The quality-adjusted life years (QALYs) and total health costs in each therapy group were assessed in 2020 Chinese yuan (¥). To facilitate case comparison, we converted the results into U.S. dollars ($) based on the exchange rate between China and the US in 2020 (i.e., $1 = ¥ 6.8974) (National Bureau of Statistics, 2020). Moreover, the incremental cost-effectiveness ratios (ICERs) were evaluated with the treatment group versus the control group. According to the recommendations of the Chinese guidelines (Liu, 2020), the model used 3% annual discount rates for costs and health outcomes and was produced from the Chinese healthcare payer perspective.

Our study’s willingness-to-pay (WTP) threshold was set to three times the value of China’s per capita GDP in 2020 ($31,512) in the base case. The model was programmed using TreeAge Pro 2019 software (TreeAge Pro Inc., Williamston, MA, United States) and was compiled with the Consolidated Health Economic Evaluation Reporting Standards (CHEERS) statement and the recent recommendations on the economic evaluation of osteoporosis (Husereau et al., 2013; Hiligsmann et al., 2019).

### Model Structure

**FIGURE 1:**
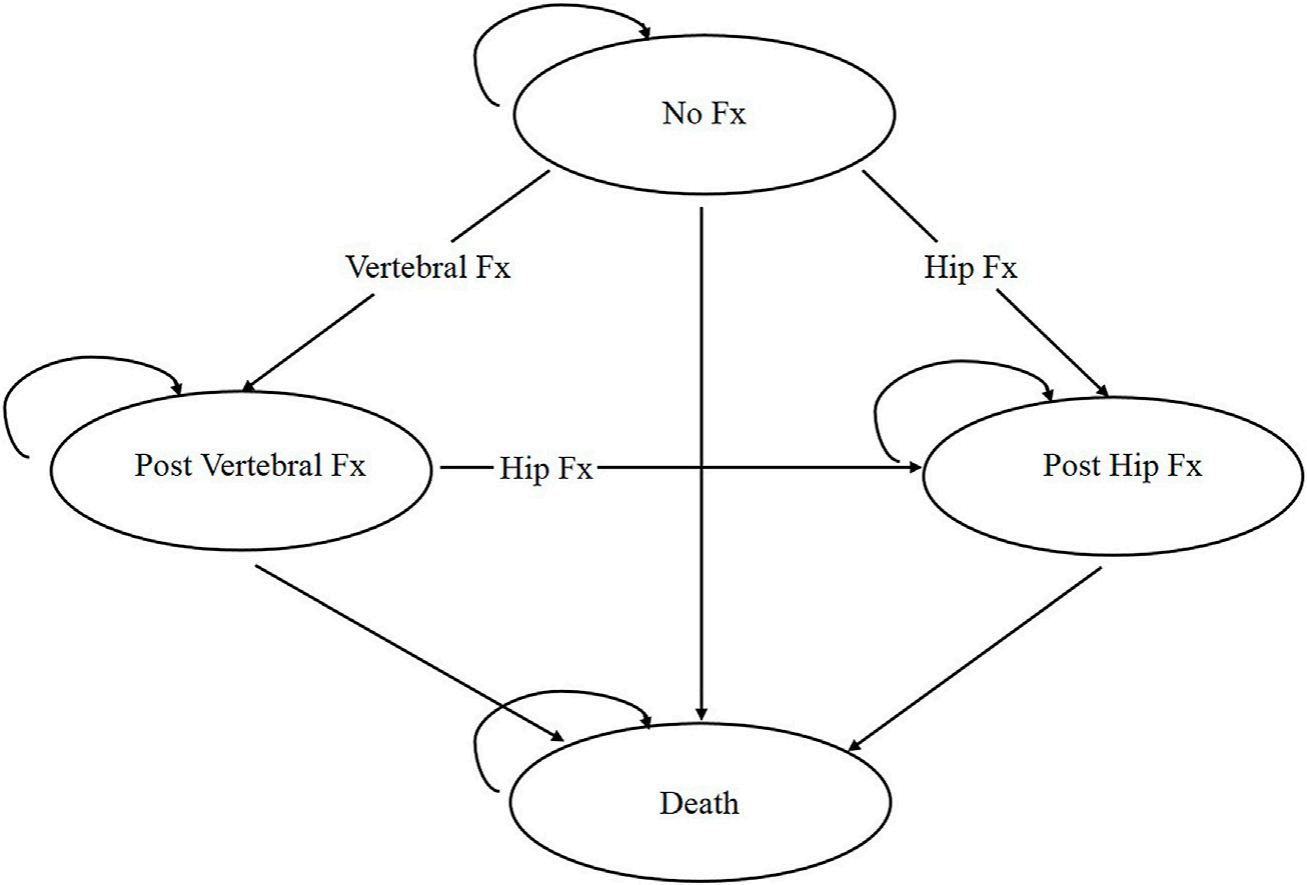
Simplified structure and transitions of the Markov model.

The simplified process framework of the model structure is shown in Figure 1. The participants’ characteristics and disease histories (e.g., number and type of fractures, time of the last fracture in the model cycle) were followed using a Markov microsimulation model. The model in our study consisted of four health states (i.e., no fracture, after clinical vertebral fracture, after hip fracture, and death). According to the participant’s Markov status, a one-time cost and disutility are allocated when the participant suffers wrist or other osteoporotic fractures. The model is set to experience only one fracture per cycle and have up to two hip fractures but an unlimited number of clinical vertebral, wrist, or other osteoporotic fractures over the entire time horizon. We showed the key critical parameters of the Markov microsimulation model in Supplementary Table S1. The specific details of the model framework can be found in the previous work of our research team (You et al., 2020; You & Liu, 2020).

### Treatment

The persistence and compliance of drugs are the key points influencing the effectiveness of treatment. Compared with oral treatment, injection treatment for osteoporosis requires less frequency of administration (You & Liu, 2020). Accordingly, the persistence and compliance of the injected drugs are more favorable, which could explain better effects for fracture prevention. Therefore, we evaluated the cost-effectiveness of sequential denosumab/zoledronic acid, which in this study was defined as subcutaneous denosumab every 6 months for 3 years followed by intravenous zoledronic acid annually for 3 years, making the total duration 6 years compared with intravenous zoledronic acid monotherapy annually for 3 years.

The efficacy and relative risk of fragility fractures with the treatments were based on the previous economic studies and recent network meta-analyses of randomized controlled trials (Mori et al., 2019; Davis et al., 2020; You et al., 2020; You & Liu, 2020).

The persistence and compliance of osteoporosis medication treatments are imperfect (Wilkes et al., 2010; Soong et al., 2013). We assumed drug persistence and compliance during the treatment based on our published studies in the Chinese or Asian population (You et al., 2020; Mori et al., 2021a). Persistence rates with denosumab and zoledronic acid were higher in clinical than observational studies based on meta-analyses. We assumed the compliance rates were 100% with denosumab or zoledronic acid.

After treatment discontinuation, there is an offset time effect, that is, the benefit of fracture reduction does not stop immediately but lasts for a period of time (Hiligsmann et al., 2019). In line with the hypotheses applied in previous studies, we assumed that the offset period of denosumab was a fixed period equal to 1 year, and that of zoledronic acid was equal to their total treatment time (Hiligsmann & Reginster, 2011; Mori et al., 2017a; Davis et al., 2020). In order to maintain the simplicity of the model, we assumed that individuals who persisted in treatment during each therapy cycle would acquire benefits from fracture prevention. In this model, only those who completed denosumab for 3 years started zoledronic acid.

### Fracture Incidence and Mortality Rates

Due to the limitation of relevant data acquisition, we extracted the annual incidence rates of hip and clinical vertebral fractures from the current epidemiological studies in the Chinese population and obtained the incidence rates of wrist and other osteoporotic fractures from studies in the United States and Norway (Lofthus et al., 2008; Sun et al., 2011; Melton et al., 2014). The accuracy of fracture risk in osteoporotic women has been improved by further calibrating the method described in our previous work (Mori et al., 2017a; You et al., 2020).

Mortality rates of the age-specific general population were obtained from the China Health Statistics Yearbook (Si et al., 2015; Si et al., 2016). We assumed that the hip fracture events would cause short-term (within 1 year) and long-term (starting from the second and subsequent years for life) excess mortality after a hip fracture (Haentjens et al., 2010). We conservatively assumed that a hip fracture itself would only contribute 25% of the additional mortality, as comorbidities appear to make a substantial contribution (Kanis et al., 2003). We did not perform a hypothetical analysis of excess mortality associated with vertebral fractures (Mori et al., 2017a; Mori et al., 2017b).

### Costs

We calculated the costs of denosumab and zoledronic acid based on the market share of original brands and generic drugs in the official database of China’s Center for Drug Evaluation (CDE) and National Medical Products Administration (NMPA). The total medication costs were drug costs multiplied by their levels of persistence and compliance throughout the treatment process. We charged one dose cost for individuals who discontinued denosumab or zoledronic acid after the first dose. The measured annual costs in the first year after fractures and long-term care costs associated with hip fractures were derived from previous studies published in the Chinese background (Qu et al., 2014; Si et al., 2016). Physician visits, laboratory examinations, DXA scans, and hospitalization costs were gathered from the hospital price system, health system, or National Development and Reform Commission of China (National Development and Reform Commission and, 2020). We dilated the costs to 2020 currency by using China’s Consumer Price Index (CPI) (National Bureau of Statistics, 2020).

### Utilities

The patient’s health-related quality of life (HRQoL) at baseline is determined by the age-specific EQ-5D score of China’s National Health Service Survey (Sun et al., 2011). An application of utility multipliers in the first year after all types of fractures and in the second and subsequent years after hip and vertebral fractures can explain the HRQoL loss in fracture patients (Hiligsmann et al., 2008; Mori et al., 2017a).

### Model Simulation and Sensitivity

We carried out base case analyses including net monetary benefit (NMB) and net health benefit (NHB), one-way deterministic sensitivity, and probabilistic sensitivity analyses. A one-way deterministic sensitivity analysis was performed to evaluate the robustness of the results of a series of values of key model parameters. In a probabilistic sensitivity analysis the parameter values were randomly selected from the probability distributions of uncertain key model inputs to explore the joint uncertainty of all input parameters based on Monte-Carlo simulations (1,000 simulations and 100,000 trials per simulation). We performed a model validation by calculating mortality and fracture rates.

## Results

### Model Validation

Without an intervention, our model estimated that the probabilities of dying before 105 years were greater than 99% at each initial age (i.e., 65, 70, 75, and 80 years), which is consistent with the 2020 Chinese life table (National Health Committee of the People’s Republic of China, 2020). Besides, our research also estimated that without an intervention, the cumulative probabilities of suffering hip or clinical vertebral fracture at least once were 11.50% or 39.69%, respectively, which are similar to China’s epidemiological data (Chinese Society of Osteoporosis and Bone Mineral Research, 2017).

### Base Case Analysis

Our model calculated the total costs, QALYs, number of fractures, ICERs, NMBs, and NHBs at various initial ages. There are diversities in the evaluation results of different initial ages (Table 1). The ICER ($/QALY gained) values corresponding to different starting ages were 59,389 at age 65, 23,821 at age 70, 22,711 at age 75, and 14,027 at age 80, respectively. At age 65 years, both NMB and NHB were negative; sequential denosumab/zoledronic acid was not cost-effective compared with zoledronic acid monotherapy at the WTP threshold of $31,512/QALY. However, the contrary results appeared at the other initial ages (i.e., 70, 75, or 80); sequential denosumab/zoledronic acid was more cost-effective than zoledronic acid monotherapy.

**TABLE 1:** The results of the base case at various ages of therapy initiation.

	No treatment	DEN/ZOL	ZOL mono	DEN/ZOL vs. ZOL mono
Aged 65 years				
Total costs (2020 US Dollars)	5,880.67	5,587.84	4,993.95	593.89
Healthcare costs	5,880.67	3,614.18	3,699.63	−85.45
Treatment costs	0	1973.66	1,294.32	679.34
QALYs	9.38	9.50	9.49	0.01
Number of fractures	1.9788	1.4977	1.5268	−0.0291
ICER ($/QALY gained)				59,389.00
NMB				−278.77
NHB				−0.0100
Aged 70 years				
Total costs (2020 US Dollars)	5,628.07	5,271.58	4,795.16	476.42
Healthcare costs	5,628.07	3,324.90	3,510.92	−186.02
Treatment costs	0	1946.68	1,284.24	662.44
QALYs	7.52	7.60	7.58	0.02
Number of fractures	1.8036	1.3154	1.3650	−0.0496
ICER ($/QALY gained)				23,821.00
NMB				153.82
NHB				0.0049
Aged 75 years				
Total costs (2020 US Dollars)	5,211.39	4,736.25	4,282.04	454.21
Healthcare costs	5,211.39	2,872.08	3,001.82	−129.74
Treatment costs	0	1864.17	1,280.22	583.95
QALYs	5.88	5.95	5.93	0.02
Number of fractures	1.5858	1.1191	1.1651	−0.0460
ICER ($/QALY gained)				22,710.50
NMB				176.03
NHB				0.0100
Aged 80 years				
Total costs (2020 US Dollars)	4,514.60	4,223.00	3,802.18	420.82
Healthcare costs	4,514.6	2,439.41	2,540.24	−100.83
Treatment costs	0	1783.59	1,261.94	521.65
QALYs	4.52	4.62	4.59	0.03
Number of fractures	1.3647	0.9443	0.9950	−0.0507
ICER ($/QALY gained)				14,027.33
NMB				524.54
NHB				0.0200

ZOL MONO, zoledronic acid monotherapy; DEN/ZOL, sequential denosumab/zoledronic acid; US Dollars, United States Dollars; QALYs, quality-adjusted life years; ICER, incremental cost-effectiveness ratio; NMB, net monetary benefit; NHB, net health benefit.

### One-Way Deterministic Sensitivity Analysis

One-way sensitivity analyses showed that the most impactful parameter was the persistence rate of the medications at the age of 65 included in this study. If the persistence rate of denosumab or zoledronic acid was increased by 10%, sequential denosumab/zoledronic acid would be cost-effective at age 65 (Table 2). At other ages, the results were stable and ICERs were below the threshold of $31,512/QALY, which meant that sequential denosumab/zoledronic acid remained cost-effective (Supplementary Table S2).

**TABLE 2:** Results of one-way analyses at 65 years.

Parameters	Cost (2020 US dollars)		△C	Effectiveness (QALYs)		△E	ICER ($/QALY gained)
	DEN/ZOL	ZOL mono		DEN/ZOL	ZOL mono		
No residual effect	5,623.57	4,985.14	638.43	9.44	9.43	0.01	63,843.00
10-years time horizon	5,582.88	4,985.29	597.59	9.46	9.44	0.02	29,879.50
DEN persistence rate 10% higher	5,776.70	4,901.31	875.39	9.53	9.50	0.03	29,179.67
ZOL persistence rate 10% higher	5,608.57	5,041.35	567.22	9.53	9.51	0.02	28,361.00
Discount rates: 0	5,865.15	5,162.38	702.77	12.41	12.39	0.02	35,138.50
Discount rates: 0.05	5,457.68	4,862.29	595.39	8.15	8.14	0.01	59,539.00
Fracture costs 30% higher	5,738.41	5,159.46	578.95	9.45	9.44	0.01	57,895.00
Fracture costs 30% lower	5,415.08	4,839.42	575.66	9.50	9.49	0.01	57,566.00
Excess mortality 50% higher	5,476.79	4,924.24	552.55	9.41	9.40	0.01	55,255.00
Excess mortality 0%	5,640.05	5,030.22	609.83	9.52	9.50	0.02	30,491.50

ZOL, zoledronic acid monotherapy; DEN, denosumab; US Dollars, United States Dollars; QALYs, quality-adjusted life years; ICER, incremental cost-effectiveness ratio.

### Probabilistic Sensitivity Analysis

**FIGURE 2:**
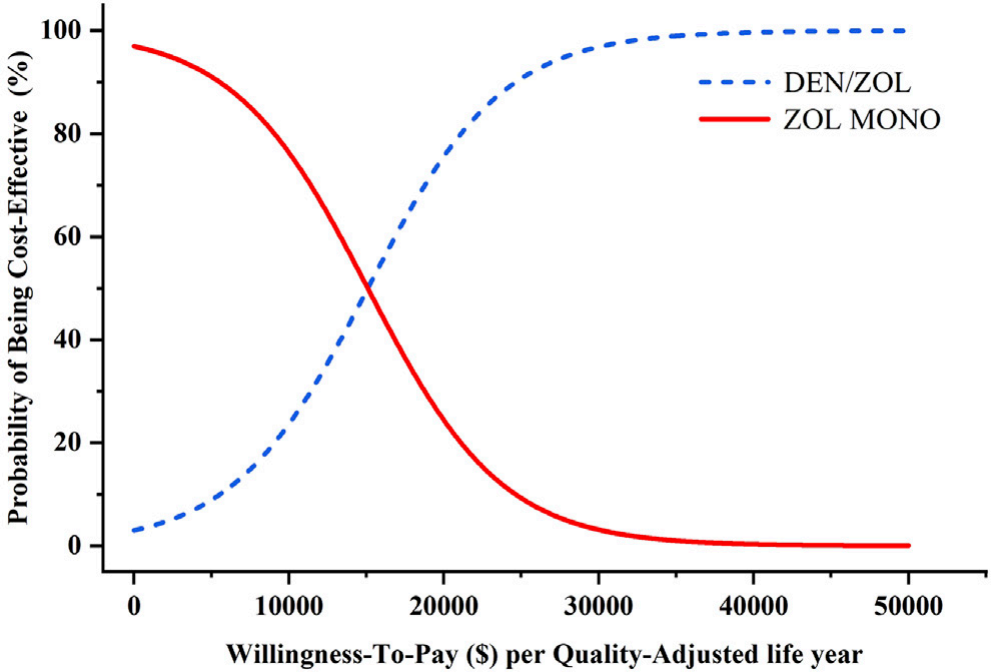
Results of probabilistic sensitivity analyses, age 80 years. The cost-effectiveness acceptability curves represent probabilities of sequential denosumab/zoledronic acid relative to zoledronic acid monotherapy being cost-effective (abbreviations: ZOL, zoledronic acid monotherapy; DEN, denosumab).

In probabilistic sensitivity analyses, the probabilities of sequential denosumab/zoledronic being cost-effective compared to zoledronic acid monotherapy were approximately 29.3%, 68.7%, 86.1%, and 99.4% for initial ages 65, 70, 75, and 80 years, respectively, at the WTP threshold of $31,512/QALY. The probability sensitivity analysis of initial age 80 was shown in Figure 2, and other ages groups were shown in Supplementary Figures S1–3.

## Discussions

We used a previously validated Markov microsimulation model to perform the economic assessment for sequential denosumab/zoledronic acid versus zoledronic acid monotherapy for postmenopausal osteoporotic women in China. Our base case analysis indicated that sequential denosumab/zoledronic acid was not cost-effective compared with zoledronic acid monotherapy at the initial age of 65 years with the WTP threshold of $31,512/QALY. However, at the starting ages of 70, 75, or 80 years old, sequential denosumab/zoledronic acid became more cost-effective than zoledronic acid monotherapy.

Cost-effectiveness analyses including zoledronic acid or denosumab for the treatment of osteoporosis have been reported. The authors from Switzerland and the United States constructed lifetime cohort Markov models and concluded that denosumab was more cost-effective or even dominant when compared with bisphosphonates in treating osteoporosis in older adults (Parthan et al., 2014; Silverman et al., 2015). Our research team previously compared the cost-effectiveness of zoledronic acid and weekly oral alendronate. We concluded that zoledronic acid was more cost-effective than oral alendronate for osteoporotic postmenopausal women without a prior fracture (You et al., 2020; You & Liu, 2020). The key parameters leading to the result were the higher persistence and compliance rates of zoledronic acid and the lower persistence rate of oral alendronate.

Oral medication is preferred for patients with low to moderate fracture risk, such as young postmenopausal women who have low bone density but no history of fractures (Feng et al., 2013; Guo et al., 2013; Eastell et al., 2019). Poor persistence and compliance, however, are common problems in the treatment of osteoporosis and they affect the ultimate effectiveness and cost. Therefore, the medications in this study are exclusively injection forms to minimize the impact of persistence and compliance on the results.

In the field of cost-effectiveness analyses, different analysis frameworks and qualifications (e.g., model parameter, time horizon, and calculations of cost and utility) lead to inconsistent conclusions (Hiligsmann et al., 2015; Li et al., 2021). The main differences of the current study compared with the previously published pharmacoeconomic analyses regarding the treatment for osteoporosis are the target population and medication dosage form. First, we focused on postmenopausal osteoporosis in China based on the fact of epidemiological studies; a large number of osteoporosis appear in postmenopausal women and the incidence rate is closely related to age. Second, we chose zoledronic acid and denosumab, which are injections and have higher persistence and compliance rates than oral medication. Denosumab has been recently marketed in China, and it is necessary to study its pharmacoeconomic in the Chinese setting.

In previous research, a Markov microsimulation was conducted to estimate the cost-effectiveness of sequential denosumab/alendronate versus zoledronic acid for postmenopausal osteoporotic women in Japan without prior fragility fracture. The results showed that zoledronic acid was cost-saving (i.e., more effective and less costly) than sequential denosumab/alendronate in the base case analyses (Mori et al., 2021b). Different from the previous study, our current study included zoledronic acid instead of oral alendronate after the completion of denosumab. Besides, our model incorporated common fractures among the target population, such as wrist and other osteoporotic fractures, into the study. A one-time cost and disutility are allocated correspondingly when the participant suffers wrist fracture or other osteoporotic fractures.

Our study’s WTP threshold was set to three times the value of China’s per capita GDP in 2020 ($31,512) in the base case. Subsequently, at the starting ages were 70, 75, or 80 years old, sequential denosumab/zoledronic acid was more cost-effective than zoledronic acid monotherapy. It is, however, important to note that sequential denosumab/zoledronic acid was not cost-effective if the WTP threshold was set to one time the value of China’s per capita GDP in 2020 ($10,504).

Several limitations in our current pharmacoeconomics analysis were noted. First, our results should be best applicable to Chinese postmenopausal women and generalized conservatively to women of other countries or races or men. The major issue regarding generalizability is the inhomogeneity of specific country settings and payer perspective. Second, although most of the data in the model were based on the Chinese context, there are still some parameters from other countries. Future cost-effectiveness evaluations should be conducted and updated when these parameter inputs are available in the Chinese context. Third, to date, there has not been a published work of a randomized controlled trial that included sequential denosumab/zoledronic acid and zoledronic acid monotherapy. Therefore, the results in the current study should be interpreted with caution. However, theoretical models are commonly used to assess the cost-effectiveness of two treatment options that are not directly compared within the same clinical trial. Fourth, the persistence rate of denosumab or zoledronic acid is a crucial parameter of this study. However, in the Chinese context, these parameters of current data were restricted. Finally, although we did not include potential adverse events in this study to keep our model concise, it is currently considered that serious adverse event caused by osteoporosis treatment strategies is rare (Chinese Society of Osteoporosis and Bone Mineral Research, 2019). So we have no strong reason to believe they have an important influence on our study.

Despite these limitations, this research also has some remarkable strengths. First, to the best of our knowledge, this is the first study to evaluate the cost-effectiveness of sequential denosumab/zoledronic acid versus zoledronic acid monotherapy for postmenopausal osteoporotic. Second, we incorporated persistence and compliance into our economic analysis and investigated how they affect results, as poor persistence and compliance have been essential parameters in cost-effectiveness analyses for current osteoporosis therapies.

In conclusion, among postmenopausal osteoporotic women older than 70 years old in China, sequential denosumab/zoledronic acid (i.e., biannual subcutaneous denosumab for 3 years followed by intravenous zoledronic acid annually for 3 years) was cost-effective compared with annual intravenous zoledronic acid monotherapy for 3 years at the pre-determined WTP threshold of $31,512/QALY. This research provides an applicable view for policymakers and clinicians from the perspective of economics regarding osteoporosis treatment in older Chinese women.

## Data Availability

The original contributions presented in the study are included in the article/Supplementary Material, further inquiries can be directed to the corresponding author.

## Supplementary Material

Supplementary Figure S1 Results of Probabilistic Sensitivity Analyses, age 65 years.

Supplementary Figure S2 Results of Probabilistic Sensitivity Analyses, age 70 years.

Supplementary Figure S3 Results of Probabilistic Sensitivity Analyses, age 75 years.

Supplementary Table S1 Summary of key parameters in the model.

Supplementary Table S2 Results of one-way analyses at 70, 75, and 80 years.
