# Supplementary material for: Cost-Effectiveness of Sequential Denosumab/Zoledronic Acid Compared With Zoledronic Acid Monotherapy for Postmenopausal Osteoporotic Women in China
Source: Front Pharmacol. 2022 Mar 18;13:816248. doi: 10.3389/fphar.2022.816248 (PMC8971554; doi:10.3389/fphar.2022.816248)
Supplement: Supplementary file 1 [file Table1.docx]

**Supplementary Table 1. Summary of key parameters in the model**

| **Parameter** | **Value** | **Range** | **Distribution** | **Reference** |
| --- | --- | --- | --- | --- |
| Denosumab therapy |  |  |  |  |
| Relative risk of hip fracture | 0.56 | 0.31-0.94 | Beta | (Davis et al., 2020) |
| Relative risk of clinical vertebral fracture | 0.30 | 0.21-0.43 | Beta | (Davis et al., 2020) |
| Relative risk of wrist fracture | 0.80 | 0.67-0.95 | Beta | (Hiligsmann & Reginster, 2011) |
| Relative risk of other osteoporotic fracture | 0.80 | 0.67-0.95 | Beta | (Hiligsmann & Reginster, 2011) |
| Adherence rate (first year) | 1.00 | N/A | N/A | (Mori et al., 2021b) |
| Persistence rate (first year) | 0.81 | 0.76-0.85 | Beta | (Mori et al., 2021b) |
| Treatment duration (years) | 3 | N/A | N/A | (Chinese Society of Osteoporosis and Bone Mineral Research, 2019) |
| Offset effect (years) | 1 | N/A | N/A | (Davis et al., 2020) |
|  |  |  |  |  |
| Zoledronic acid therapy |  |  |  |  |
| Relative risk of hip fracture | 0.64 | 0.47-0.86 | Beta | (Davis et al., 2020) |
| Relative risk of clinical vertebral fracture | 0.40 | 0.29-0.55 | Beta | (Davis et al., 2020) |
| Relative risk of wrist fracture | 0.75 | 0.64-0.87 | Beta | (You et al., 2020) |
| Relative risk of other osteoporotic fracture | 0.69 | 0.55-0.84 | Beta | (You et al., 2020) |
| Adherence rate (first year) | 1.00 | N/A | N/A | (You et al., 2020) |
| Persistence rate (First year) | 1.00 | N/A | Triangular | (You et al., 2020) |
| Treatment duration (years) | 3 | N/A | N/A | (Chinese Society of Osteoporosis and Bone Mineral Research, 2019) |
| Offset effect (years) | 3 | N/A | N/A | (Davis et al., 2020) |
|  |  |  |  |  |
| Costs (2020 US dollars) |  |  |  |  |
| Annual cost for denosumab | 180.80 | 126.56-235.04 | Triangular | (National Medical Products Administration; Center for Drug Evaluation, NMPA) |
| Annual cost for zoledronic acid | 369.01 | 258.31-479.71 | Triangular | (National Medical Products Administration; Center for Drug Evaluation, NMPA) |
| Hip fracture, medical costs | 7306.75 | 5114.73-9498.78 | Triangular | (Si et al., 2016) |
| Clinical vertebral fracture, medical costs | 1347.64 | 943.35-1751.94 | Triangular | (Si et al., 2016) |
| Wrist fracture, medical costs | 995.05 | 696.54-1293.57 | Triangular | (Si et al., 2016) |
| Other osteoporotic fracture, medical costs | 1740.89 | 1218.63-2263.17 | Triangular | (Si et al., 2016) |
| Annual long-term care costs for the post-hip fracture state | 4565.23 | 3195.66-5934.80 | Triangular | (Si et al., 2016) |
| DEXA scan | 87.44 | 61.20-113.67 | Triangular | (National Medical Products Administration) |
| Blood test | 74.06 | 51.84-96.28 | Triangular | (National Medical Products Administration) |
| Physician visit | 10.29 | 7.20-13.37 | Triangular | (National Medical Products Administration) |
|  |  |  |  |  |
| Utilities |  |  |  |  |
| Age 65-69 (baseline) | 0.806 | 0.765-0.846 | Beta | (Sun et al., 2011) |
| Age 70-74 (baseline) | 0.747 | 0.709-0.784 | Beta | (Sun et al., 2011) |
| Age 75-79 (baseline) | 0.731 | 0.694-0.767 | Beta | (Sun et al., 2011) |
| Age 80-84 (baseline) | 0.699 | 0.664-0.733 | Beta | (Sun et al., 2011) |
| Age 85+ (baseline) | 0.676 | 0.642-0.709 | Beta | (Sun et al., 2011) |
| Hip fracture, first year (multiplier) | 0.776 | 0.720-0.844 | Beta | (Si, Winzenberg, de Graaff, & Palmer, 2014) |
| Hip fracture, subsequent year (multiplier) | 0.855 | 0.800-0.909 | Beta | (Si, Winzenberg, de Graaff, & Palmer, 2014) |
| Clinical vertebral fracture, first year (multiplier) | 0.724 | 0.667-0.779 | Beta | (Si, Winzenberg, de Graaff, & Palmer, 2014) |
| Clinical vertebral fracture, subsequent year (multiplier) | 0.868 | 0.827-0.922 | Beta | (Si, Winzenberg, de Graaff, & Palmer, 2014) |
| Wrist fracture (multiplier) | 0.940 | 0.910-0.960 | Beta | (Hiligsmann et al., 2008) |
| Other osteoporotic fracture (multiplier) | 0.910 | 0.880-0.940 | Beta | (Hiligsmann, Ethgen, Richy, & Reginster, 2008) |
|  |  |  |  |  |
| Annual fracture incidence per 1000 persons (without an intervention) | | | | |
| Hip fracture, age 65-69 | 0.96 | N/A | N/A | (J. Wang, Wang, Liu, Wang, & Yin, 2014) |
| Hip fracture, age 70-74 | 2.33 | N/A | N/A | (Wang, Wang, Liu, Wang, & Yin, 2014) |
| Hip fracture, age 75-79 | 4.08 | N/A | N/A | (Wang, Wang, Liu, Wang, & Yin, 2014) |
| Hip fracture, age 80-84 | 6.44 | N/A | N/A | (Wang, Wang, Liu, Wang, & Yin, 2014) |
| Hip fracture, age 85+ | 6.59 | N/A | N/A | (Wang, Wang, Liu, Wang, & Yin, 2014) |
|  |  |  |  |  |
| Clinical vertebral fracture, age 65-69 | 5.64 | N/A | N/A | (Bow et al., 2012) |
| Clinical vertebral fracture, age 70-74 | 8.74 | N/A | N/A | (Bow et al., 2012) |
| Clinical vertebral fracture, age 75-79 | 12.05 | N/A | N/A | (Bow et al., 2012) |
| Clinical vertebral fracture, age 80-84 | 21.19 | N/A | N/A | (Bow et al., 2012) |
| Clinical vertebral fracture, age 85+ | 26.89 | N/A | N/A | (Bow et al., 2012) |
|  |  |  |  |  |
| Wrist fracture, age 65-69 | 12.95 | N/A | N/A | (Lofthus et al., 2008) |
| Wrist fracture, age 70-74 | 13.17 | N/A | N/A | (Lofthus et al., 2008) |
| Wrist fracture, age 75-79 | 13.87 | N/A | N/A | (Lofthus et al., 2008) |
| Wrist fracture, age 80-84 | 15.01 | N/A | N/A | (Lofthus et al., 2008) |
| Wrist fracture, age 85+ | 15.10 | N/A | N/A | (Lofthus et al., 2008) |
|  |  |  |  |  |
| Other osteoporotic fracture, age 65-69 | 6.60 | N/A | N/A | (Mori et al., 2017a) |
| Other osteoporotic fracture, age 70-74 | 9.84 | N/A | N/A | (Mori, Crandall, & Ganz, 2017) |
| Other osteoporotic fracture, age 75-79 | 14.44 | N/A | N/A | (Mori, Crandall, & Ganz, 2017) |
| Other osteoporotic fracture, age 80-84 | 18.06 | N/A | N/A | (Mori, Crandall, & Ganz, 2017) |
| Other osteoporotic fracture, age 85+ | 26.06 | N/A | N/A | (Mori, Crandall, & Ganz, 2017) |
|  |  |  |  |  |
| Relative risks of fractures for those with osteoporosis | | | | |
| Hip fracture, age 65-69 | 3.91 | 3.28-4.56 | Gamma | (Johnell et al., 2005; Kanis et al., 2000) |
| Hip fracture, age 70-74 | 3.13 | 2.80-3.47 | Gamma | (Johnell et al., 2005; Kanis et al., 2000) |
| Hip fracture, age 75-79 | 2.60 | 2.39-2.82 | Gamma | (Johnell et al., 2005; Kanis et al., 2000) |
| Hip fracture, age 80-84 | 2.04 | 1.91-2.17 | Gamma | (Johnell et al., 2005; Kanis et al., 2000) |
| Hip fracture, age 85+ | 1.92 | 1.78-2.05 | Gamma | (Johnell et al., 2005; Kanis et al., 2000) |
|  |  |  |  |  |
| Clinical vertebral fracture, age 65-69 | 2.59 | 1.19-4.27 | Gamma | (Kanis et al., 2000; Marshall et al., 1996) |
| Clinical vertebral fracture, age 70-79 | 2.15 | 1.15-3.15 | Gamma | (Kanis et al., 2000; Marshall et al., 1996) |
| Clinical vertebral fracture, age 80+ | 1.82 | 1.12-2.41 | Gamma | (Kanis et al., 2000; Marshall et al., 1996) |
|  |  |  |  |  |
| Wrist fracture, age 65-69 | 1.78 | 1.78-2.19 | Gamma | (Kanis et al., 2000; Marshall et al., 1996) |
| Wrist fracture, age 70-79 | 1.60 | 1.60-1.88 | Gamma | (Kanis et al., 2000; Marshall et al., 1996) |
| Wrist fracture, age 80+ | 1.45 | 1.45-1.64 | Gamma | (Kanis et al., 2000; Marshall et al., 1996) |
|  |  |  |  |  |
| Other osteoporotic fracture, age 65-69 | 2.19 | 1.78-2.59 | Gamma | (Kanis et al., 2000; Marshall et al., 1996) |
| Other osteoporotic fracture, age 70-79 | 1.88 | 1.60-2.15 | Gamma | (Kanis et al., 2000; Marshall et al., 1996) |
| Other osteoporotic fracture, age 80+ | 1.64 | 1.45-1.82 | Gamma | (Kanis et al., 2000; Marshall et al., 1996) |
|  |  |  |  |  |
| Annual mortality rate |  |  |  |  |
| 65-69 | 0.01031 | N/A | N/A | (Si et al., 2016) |
| 70-74 | 0.02036 | N/A | N/A | (Si et al., 2016) |
| 75-79 | 0.03784 | N/A | N/A | (Si et al., 2016) |
| 80-84 | 0.06998 | N/A | N/A | (Si et al., 2016) |
| 85+ | 0.13603 | N/A | N/A | (Si et al., 2016) |
|  |  |  |  |  |
| Excess mortality after a hip fracture |  |  |  |  |
| Relative hazard for mortality within a year after a hip fracture | 2.87 | 2.52-3.27 | N/A | (Haentjens et al., 2010) |
| Relative hazard for mortality for second and beyond after a hip fracture | 1.73 | 1.56-1.90 | N/A | (Haentjens et al., 2010) |
| The proportion of excess mortality after a hip fracture directly attributable to a hip fracture | 0.25 | N/A | N/A | (Kanis et al., 2003) |
|  |  |  |  |  |
| Discount rates |  |  |  |  |
| Costs | 0.03 | 0-0.05 | Triangular | (You & Liu, 2020) |
| Effectiveness | 0.03 | 0-0.05 | Triangular | (You & Liu, 2020) |
