# Supplementary figures and images for: Cost-Effectiveness of Sequential Denosumab/Zoledronic Acid Compared With Zoledronic Acid Monotherapy for Postmenopausal Osteoporotic Women in China
Source: Front Pharmacol. 2022 Mar 18;13:816248. doi: 10.3389/fphar.2022.816248 (PMC8971554; doi:10.3389/fphar.2022.816248)

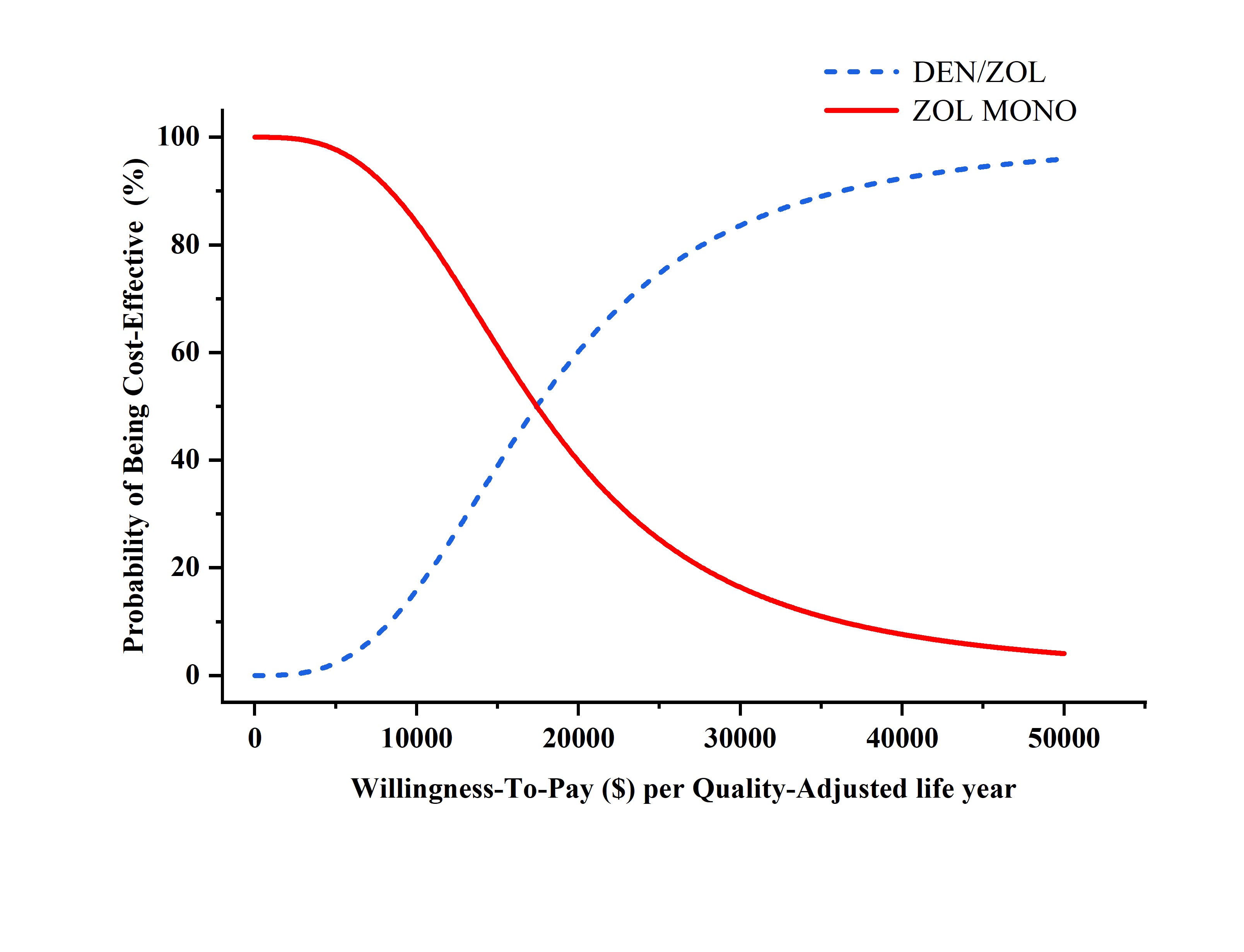

Supplement: Supplementary file 2 [file Image3.TIF]

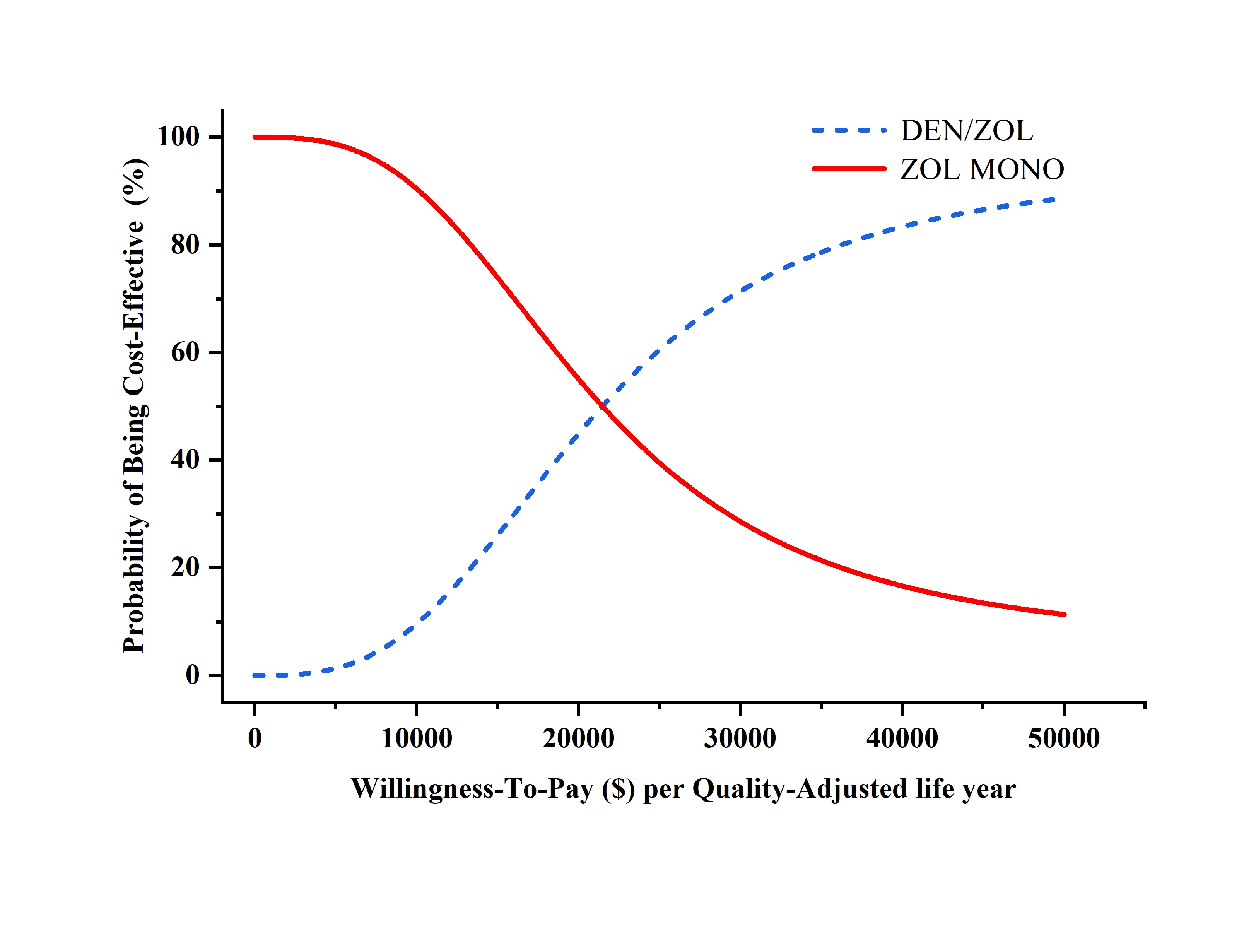

Supplement: Supplementary file 3 [file Image2.TIF]

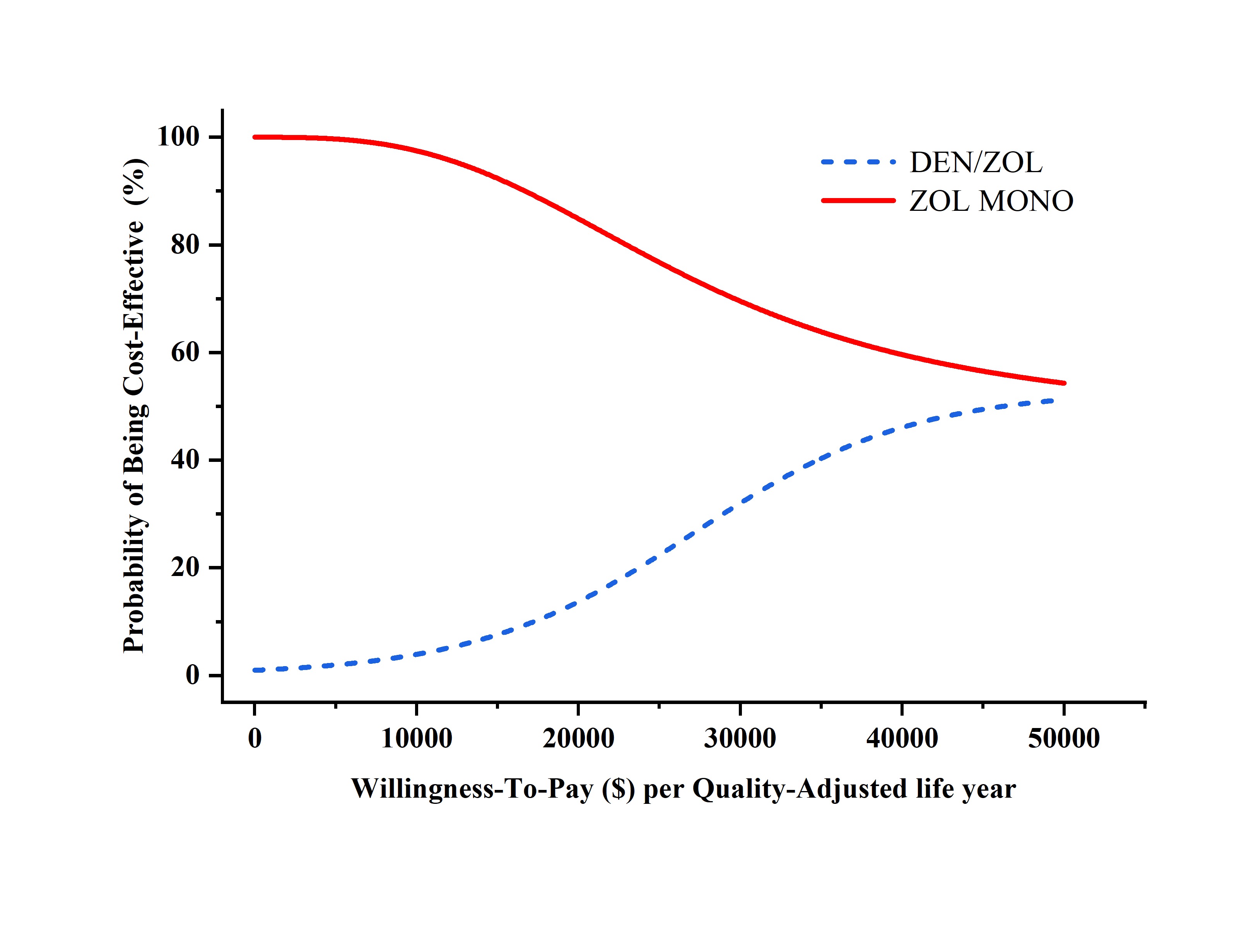

Supplement: Supplementary file 4 [file Image1.TIF]
