# Supplementary material for: Cost-Effectiveness of Sequential Denosumab/Zoledronic Acid Compared With Zoledronic Acid Monotherapy for Postmenopausal Osteoporotic Women in China
Source: Front Pharmacol. 2022 Mar 18;13:816248. doi: 10.3389/fphar.2022.816248 (PMC8971554; doi:10.3389/fphar.2022.816248)
Supplement: Supplementary file 5 [file Table2.docx]

**Supplemental Table 2. Results of one-way analyses at 70, 75, and 80 years**

| **Parameters** | **Cost** | | **△C** | **Eff** | | **△E** | **ICER** |
| --- | --- | --- | --- | --- | --- | --- | --- |
|  | DEN/ZOL | ZOL MONO |  | DEN/ZOL | ZOL MONO |  |  |
| **Aged 70 years** |  |  |  |  |  |  |  |
| No residual effect | 5343.84 | 4731.09 | 612.75 | 7.58 | 7.56 | 0.02 | 30637.5 |
| 10-year time horizon | 5175.87 | 4585.43 | 590.44 | 7.54 | 7.52 | 0.02 | 29522 |
| DEN persistence rate 10% higher | 5321.09 | 4611.77 | 709.32 | 7.62 | 7.59 | 0.03 | 23644 |
| ZOL persistence rate 10% higher | 5258.26 | 4810.16 | 448.1 | 7.63 | 7.61 | 0.02 | 22405 |
| Discount rates: 0 | 5547.59 | 4920.4 | 627.19 | 9.54 | 9.52 | 0.02 | 31359.5 |
| Discount rates: 0.05 | 5000.76 | 4468.98 | 531.78 | 6.58 | 6.56 | 0.02 | 26589 |
| Fracture costs 30% higher | 5403.66 | 4788.75 | 614.91 | 7.59 | 7.57 | 0.02 | 30745.5 |
| Fracture costs 30% lower | 5124.12 | 4516.51 | 607.61 | 7.57 | 7.55 | 0.02 | 30380.5 |
| Excess mortality 50% higher | 5509.19 | 4857.17 | 652.02 | 7.56 | 7.54 | 0.02 | 32601 |
| Excess mortality 0% | 5289.17 | 4719.36 | 569.81 | 7.62 | 7.6 | 0.02 | 28490.5 |
| **Aged 75 years** |  |  |  |  |  |  |  |
| No residual effect | 4838.74 | 4301.55 | 537.19 | 5.94 | 5.92 | 0.02 | 26859.5 |
| 10-year time horizon | 4736.9 | 4209.38 | 527.52 | 5.91 | 5.89 | 0.02 | 26376 |
| DEN persistence rate 10% higher | 4880.8 | 4292.36 | 588.44 | 5.99 | 5.96 | 0.03 | 19614.667 |
| ZOL persistence rate 10% higher | 4758.6 | 4308.05 | 450.55 | 5.98 | 5.96 | 0.02 | 22527.5 |
| Discount rates: 0 | 5062.61 | 4529.87 | 532.74 | 7.36 | 7.34 | 0.02 | 26637 |
| Discount rates: 0.05 | 4657.28 | 4147.25 | 510.03 | 5.37 | 5.35 | 0.02 | 25501.5 |
| Fracture costs 30% higher | 4889.9 | 4423.6 | 466.3 | 5.99 | 5.97 | 0.02 | 23315 |
| Fracture costs 30% lower | 4533.75 | 4035.34 | 498.41 | 5.97 | 5.95 | 0.02 | 24920.5 |
| Excess mortality 50% higher | 4928.88 | 4361.04 | 567.84 | 5.95 | 5.93 | 0.02 | 28392 |
| Excess mortality 0% | 4787.94 | 4312.37 | 475.57 | 6.02 | 6 | 0.02 | 23778.5 |
| **Aged 80 years** |  |  |  |  |  |  |  |
| No residual effect | 4281.16 | 3841.59 | 439.57 | 4.59 | 4.57 | 0.02 | 21978.5 |
| 10-year time horizon | 4118.3 | 3709.93 | 408.37 | 4.6 | 4.58 | 0.02 | 20418.5 |
| DEN persistence rate 10% higher | 4341.03 | 3780.19 | 560.84 | 4.64 | 4.6 | 0.04 | 14021 |
| ZOL persistence rate 10% higher | 4112.84 | 3821.87 | 290.97 | 4.62 | 4.6 | 0.02 | 14548.5 |
| Discount rates: 0 | 4332.97 | 3840.74 | 492.23 | 5.44 | 5.41 | 0.03 | 16407.667 |
| Discount rates: 0.05 | 4094.37 | 3713.98 | 380.39 | 4.14 | 4.12 | 0.02 | 19019.5 |
| Fracture costs 30% higher | 4280.09 | 3868.92 | 411.17 | 4.6 | 4.58 | 0.02 | 20558.5 |
| Fracture costs 30% lower | 4044.5 | 3605.96 | 438.54 | 4.58 | 4.56 | 0.02 | 21927 |
| Excess mortality 50% higher | 4249.52 | 3826.49 | 423.03 | 4.61 | 4.59 | 0.02 | 21151.5 |
| Excess mortality 0% | 4210.25 | 3780.37 | 429.88 | 4.65 | 4.62 | 0.03 | 14329.333 |

Abbreviations: ZOL, zoledronic acid monotherapy; DEN, denosumab; US Dollars, United States Dollars; QALYs, quality-adjusted life years; ICER, incremental cost-effectiveness ratio.
